# Credibility of vaccine-related content on Twitter during COVID-19 pandemic

**DOI:** 10.1371/journal.pgph.0001385

**Published:** 2023-07-19

**Authors:** Samira Yousefinaghani, Rozita Dara, Alice Wang, Melissa MacKay, Andrew Papadopoulos, Shayan Sharif

**Affiliations:** 1 School of Computer Science, University of Guelph, Guelph, Ontario, Canada; 2 Department of Pathobiology, University of Guelph, Guelph, Ontario, Canada; 3 Department of Population Medicine, University of Guelph, Guelph, Ontario, Canada; University of New South Wales, AUSTRALIA

## Abstract

During national COVID-19 vaccine campaigns, people continuously engaged on Twitter to receive updates on the latest public health information, and to discuss and share their experiences. During this time, the spread of misinformation was widespread, which threatened the uptake of vaccines. It is therefore critical to understand the reasons behind vaccine misinformation and strategies to mitigate it. The current research aimed to understand the content of misleading tweets and the characteristics of their corresponding accounts. We performed a machine learning approach to identify misinformation in vaccine-related tweets, and calculated the demographic, engagement metrics and bot-like activities of corresponding accounts. We found critical periods where high amounts of misinformation coincided with important vaccine announcements, such as emergency approvals of vaccines. Moreover, we found Asian countries had a lower percentage of misinformation shared compared to Europe and North America. Our results showed accounts spreading misinformation had an overall 10% greater likelihood of bot activity and 15% more astroturf bot activity than accounts spreading accurate information. Furthermore, we found that accounts spreading misinformation had five times fewer followers and three times fewer verified badges than fact-sharing accounts. The findings of this study may help authorities to develop strategies to fight COVID-19 vaccine misinformation and improve vaccine uptake.

## 1. Introduction

The continuous exposure to a highly connected online world, social media, and the circulation of misinformation through social media can influence users to share and communicate inaccurate information [1]. Misinformation is defined as the spread of false or inaccurate information unknowingly, whereas disinformation is spreading inaccurate information knowingly [2]. Misinformation can also be described as misleading statements without evidence or known origin [3]. Vaccine misinformation can include damaging health advice, promotion of dangerous treatments, anti-vaccine statements, and politically-motivated conspiracy theories [4–6].

A study in the United Kingdom showed 40% of adults encountered negative vaccine-related information on social media platforms [7]. Moreover, findings by Stecula and colleagues [8] indicated that individuals were more likely to be misinformed about vaccines on social media than traditional media sources. The spread of misinformation on social media can make it challenging for the public to find trustworthy sources and reliable guidance [9].

Despite the proven effectiveness and safety of COVID-19 vaccines, people might refuse these vaccines or delay taking them due to low confidence [10]. This can lead to interruption in disease control plans and potentially threaten public health [11, 12]. Therefore, as the interest in using social media enhances, professionals in the health sector should make efforts to fight against COVID-19 vaccine misinformation. In order for vaccine plans to be successful, it is necessary to track the spread of vaccine misinformation on social media [13, 14]. Understanding the drivers of misinformation dissemination on social media can then maximize vaccine acceptance. To achieve this objective, the first step is to identify misinformation in the massive amount of data that are generated in social media. Machine learning and natural language processing can be used for this purpose as they have been proven to be effective in the automatic detection of misinformation and fake news [15].

Several approaches have been used to identify misinformation content on social media. Al-Rakhami and Al-Amri [2] utilized user and tweet-level features and conducted a supervised classification to find credible COVID-19 information on Twitter. In addition to social media, the credibility of news items has been also investigated. Ahmad and colleagues [16] proposed an ensemble learning approach to detect fake news and discovered textual patterns that could distinguish fake and true news articles. Ensemble learning combines useful insights from multiple learning algorithms that individually produce weaker results using voting methods [17]. Compared to single models, these evaluations showed a higher performance of the ensemble learning.

The process of manual categorization of tweets into credible and non-credible in the literature was completed using annotation guidelines and reliable sources that were provided to human annotators [2]. Similarly, credible domain datasets and fact-checking websites were used in other studies [18, 19].

Several studies developed fact checking datasets such as a multimodal (images, text and temporal information) COVID-19 vaccine focused data repository consisting of 2,593 news articles [20]. Approaches such as iterative query selection (IQS) algorithm were used to improve information retrieval by interacting with search engines [21]. IQS algorithm was used to automatically collect a large-scale dataset of true and the fake news items for fake news detection task.

Identifying socio-demographic factors contributing to vaccine misinformation, followed by targeting and tailoring public health messaging to combat misinformation can improve vaccine uptake [22]. Survey research has been conducted to analyze the impact of sociodemographic factors such as age, gender, education, and ethnicity on the acceptance of vaccines [23, 24].

The aim of the present study was to examine COVID-19 vaccine misinformation on Twitter to understand the context and spreaders’ accounts in terms of demographic information, bot activities, and engagement metrics. The insights obtained from this study may help improve the management of communication surrounding vaccination programs and to enhance the acceptance of message by public health and uptake of vaccination. The main objectives of the current study were as follows: (1) to build a misinformation prediction model at the time of pandemic using machine learning and a combination of labelled and unlabelled data; (2) to find the timeline and geographical distribution of misinformation tweets; and (3) to identify demographic, engagement metrics, and bot activities of misinformation-spreaders’ accounts.

The first novelty in the present study was the automatic large-scale identification of vaccine misinformation tweets to help demonstrate the progression of misinformation across countries. We selected a critical period between November 2020 to May 2021, when vaccine candidates were approved for emergency use and distribution. Understanding the progression of misinformation content is valuable as it helps identify points of time in each country when false content had increased. Recognizing the reason behind a surge in misinformation content in a specific country can help predict critical points, and will consequently prepare public health authorities, the scientific community, and politicians to fight misinformation in future vaccination programs.

The second novelty lies in finding the characteristics of Twitter accounts that were involved in sharing misinformation. In addition to identifying demographic information of these accounts, we found their verified badges, engagement metrics, and bot activities. Previous survey studies [24, 25] have investigated the impact of some socio-demographic characteristics on vaccine acceptance and hesitancy, but the impact of factors such as organization status, verified status, engagement metrics, and bot activities is still poorly understood. Making sense of the demographic and characteristics of misinformation spreaders in an automatic manner can facilitate choosing the target population for suspension or educational purposes. Targeted crisis communication on social media can create an interactive dialogue where questions can be answered, which would positively impact message acceptance and vaccine uptake.

## 2. Materials and methods

**Fig 1:**
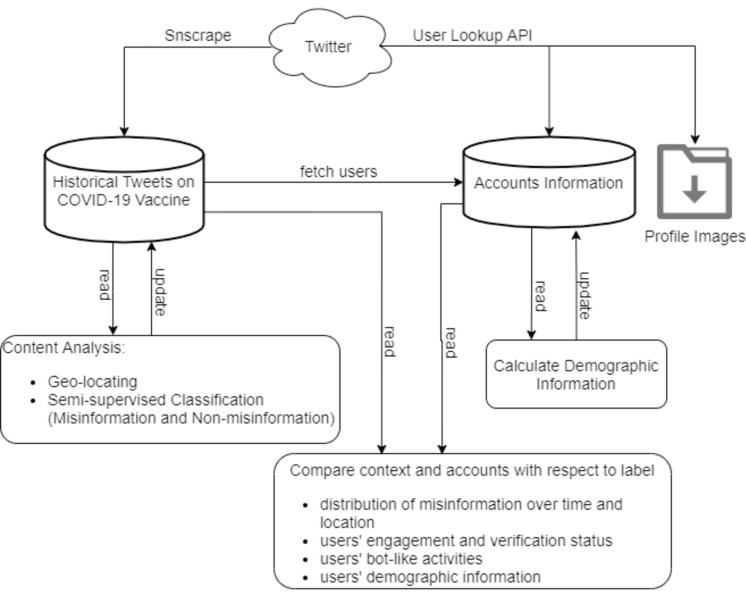
Overall methodology of machine learning model to predict COVID-19 misinformation.

The overall approach of the study can be found in Fig 1. We collected COVID-19 vaccine related tweets and their associated users’ information from November 2020 to May 2021. A semi-supervised model was used to learn a classification function to predict unlabeled tweets to information (facts) and misinformation (false) class labels. Additionally, users’ profile locations were converted to standard country names. Subsequently, the progression of misinformation was plotted to understand how it propagated based on critical events and geographical locations. Subsequently, the demographic information of users including age intervals (≤ 18, 19–29, 30–39, ≥ 40), organization status (org, non-org) and gender (male, female) was calculated. We then investigated the impact of user characteristics, engagement metrics and demographic attributes on the spread of misinformation (Fig 1).

### 2.1 Tweet acquisition

A scraper for social networking services [26] was utilized to collect tweets related to the COVID-19 vaccination (Fig 1). Terms related to COVID-19 such as “covid”, “coronavirus”, “ncov2019” and “SARS-CoV-2” were combined with “vaccine” term as input of the scraper. In total, 7,235,159 tweets generated by 2,223,969 users were retrieved between November 2020 and May 2021, when Twitter was dominated by the news of the COVID-19 pandemic ending through vaccine distribution. The ‘tweets’ data table included following fields: ‘id’, ‘date’, ‘tweet’, ‘url’, ‘username’, ‘outlinks’, ‘retweetCount’, ‘replyCount’, ‘likeCount’ and ‘quoteCount’ for describing a tweet entity.

### 2.2 Users’ information acquisition

The user lookup Twitter API was employed to gather information of users that authored the collected vaccine-related tweets. Users’ information including ‘id’, ‘screenName’, ‘name’, ‘profileLocation’, ‘description’, ‘followersCount’, ‘friendsCount’, ‘favouritesCount’, and ‘verified’ were stored in a table and profile images of collected users were downloaded to be used in further analyses (Fig 1). Verified status shows that an account is not run by bots or spammers and provides authentic value and credibility [10]. Accessing standardized geographical names was necessary for further analyses. Therefore, we added an extra table field (country) in user tables and applied SQL update queries to map self-reported profile locations to country names. Mapping was performed using a set of pre-defined geographical terms for each country, including countries, regions, provinces, and city names [27].

### 2.3 Users’ demographic information

We sought to understand which specific groups of users hold misinformation beliefs. Identifying demographic attributes that distinguish individuals who spread misleading content from others can help profile a specific group of accounts to be targeted for limited activities or suspension. The M3-inference model [28] was applied to infer the demographic information of all unique Twitter users that created tweets used in the present study (Fig 1). M3-inference, with a multimodal deep neural architecture and multi-language support, performs a joint classification of age, gender, and organization/non-organization status given users’ name, screen name, profile description and profile image. The org/non-org status means if a user account is an individual account or it corresponds to an organization. Two separate pipelines are included for co-training of a profile image and three texts. Image classification uses DenseNet model [29] and text model uses 2-stack bidirectional Long-short Term Memory (LSTM) [30].

The M3-inference model was tested by [28] against several similar systems to evaluate the performance of each module. The M3-inference model substantially outperforms or shows similar performance to existing systems. In addition, Wang and colleagues [28] found the model is accurate enough in the multilingual European environment. Due to the computational costs of the process, the demographic information for 10% of total users was analysed. In our study, we adopted a PyTorch implementation of the M3-inference which uses a pre-trained model that was trained on a massive Twitter dataset.

### 2.4 Misinformation identification

Exposure to misinformation stories around COVID-19 on Twitter can affect the public’s’ intent to receive vaccines [31]. Detecting misinformation content on Twitter is the first step to understanding the drivers of vaccine misinformation and combat it. To this end, a machine-learning model was used to identify tweets containing vaccine misinformation (Fig 1). Initially, approximately 5,000 tweets were manually categorized by an annotator into 1 and 0, indicating a tweet is reporting misinformation or not, respectively [32, 33]. Table 1 shows a list of example criteria that were used as a guideline to identify the labels. A complete list of criteria is given in S1 Table. Subsequently, the results were reviewed by the second annotator and labels were modified when necessary. Among the labeled instances, initially, 4,395 were assigned with a target value of 0 and the rest with 1 (i.e., 605 misinformation tweets).

**Table 1:** Examples of labelling criteria.

misinformation	non-misinformation
Suggesting treatments without any credible source of information: for example,”taking Zink is more effective than experimental vaccines” or”I don’t need vaccine because I already got COVID and I have anti-bodies”.	individuals’ vaccination experience.
Blames about vaccine consequences without a credible source: for example,”Moderna vaccine can mess you up”	hopes and prayers for the end the pandemic with vaccines. advertisement. political issues.

To address the class imbalance in the training dataset, we enhanced the labeled dataset by 4,000 using a SQL query to filter tweets related to misinformation (label 1). Adding extra tweets resulted in 4,395 tweets with label 0 and 4,605 tweets with label 1. The oversampling helps to increase the impact of the minority class and reduce information loss. Terms such as “5G”, “poison”, “microchip”, “hoax” and other words that are indicators of misinformation were included in the query. Tweets containing terms such as ‘misleading’ and conspiracy were excluded as they were tweets informing and warning users about COVID-19 misinformation. For example, the tweet ‘*The conspiracy theorists believe that if you have the new COVID vaccine*, *your body will turn into a 5G antenna*’ is about 5G but does not convey a misinformation message. We reviewed the query output to ensure its accuracy, and when necessary, the irrelevant tweets were discarded. The terms used in the query were selected based on a random sample of tweets and then the outcome tweets were checked to ensure their relevance to misinformation stories.

**Fig 2:**
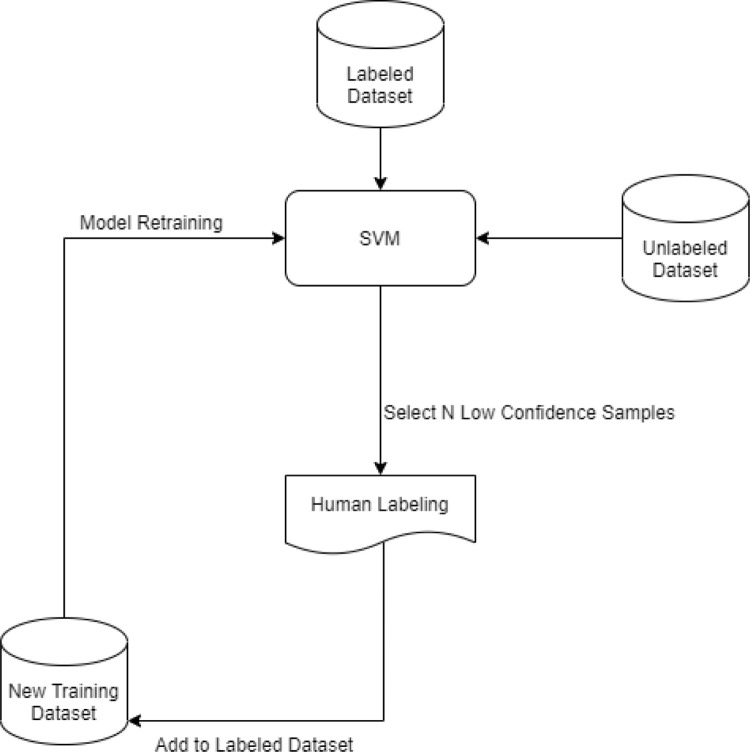
Learning pipeline: Labeling tweets to misinformation or fact.

The active-learning approach was used to reduce the time and expense related to obtaining labeled examples. The learning algorithm in active learning iteratively selects the most informative instances from unlabeled data and asks a human expert for the correct labels. The most informative instances are those that the learner algorithm is least confident about their labels (Fig 2). Subsequently, the human-labelled instances are added to the training set and this procedure continues until a stopping criterion is met [34].

Since our research required all data instances to be labeled as one of the target groups (i.e., misinformation and fact), we adopted an active-learning pipeline. We chose Linear Support Vector Classification (LinearSVC), which is an inbuilt variation of a Support Vector Machine (SVM), to train the model on a dataset of 9,000 tweets. Convenient mathematical properties of SVM classifiers make them suitable for active learning [35]. A randomized cross-validation was employed to calculate the average performance metrics. After each evaluation, the model was re-trained in an iterative procedure until the stopping criterion, i.e., 50 iterations, was reached. In each iteration, 100 samples with the lowest average absolute confidence were selected to be labeled by experts. The selection was based on the decision function of the SVM algorithm, which is defined as the distance of the samples X to the separating hyperplane. Following that, the labeled samples were removed from the unlabeled pool and were added to the training set. The final model obtained an accuracy of 92% and a F-score of 87%.

### 2.5 Bot-like accounts identification

We utilized an online service called Botometer (formerly BotOrNot) which uses Twitter Application Programming Interface (API) and was implemented via RapidAPI service. The Botometer has shown accurate results examining the likelihood of a Twitter account to be a bot and identifying a bot-like behaviour [36–38].

The Botometer service uses machine learning and analyses features, content, sentiments, network structure, user metadata, and user temporal activities to calculate scores. The aggregated score of this process is between 0 and 1, indicating the likelihood that the account belongs to a bot, when low scores indicate a likely human account while high scores indicate a likely bot account.

## 3. Results

A total of 7,235,182 tweets were collected between November 2020 and May 2021, created by 2,223,182 unique users. Six percent of the total posts were classified as misinformation created by 284,426 unique users.

### 3.1 Misinformation by time

**Fig 3:**
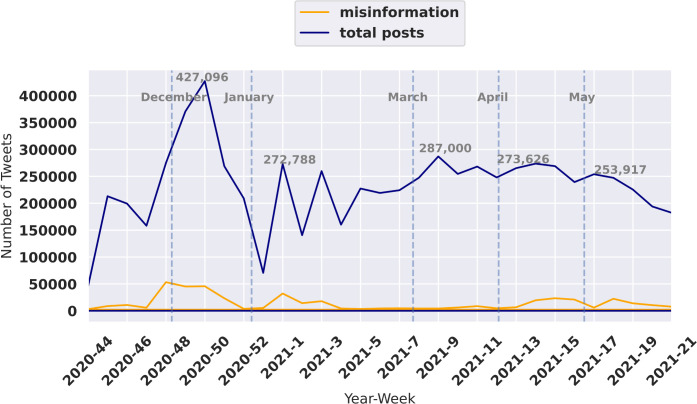
Total tweets and misinformation timelines. The x-axis shows the time period between November, denoting with 2020–44 (week 44 of year 2020), and the end of May (week 21 of year 2021). The y-axis shows the number of tweets posted per week.

Understanding the progression of misinformation by time and location is useful in finding the underlying reasons behind spikes in misinformation content. The weekly time-series of total collected COVID-19 vaccine-related tweets and COVID-19 vaccine misinformation over seven months is depicted in Fig 3.

The number of tweets reached approximately 22,000 tweets in November and increased to its highest point with about 427,096 tweets in December. The increase in vaccine related discussions and misinformation coincided with The United States Food and Drug Administration issuing Emergency Use Authorization (EUA) for Moderna COVID-19 vaccine on December 11 followed by the approval of EUA for Moderna COVID-19 vaccine on December 18 (FDA (2020)). The number of related tweets fluctuated between 70,000 and 287,000 after December with the highest number of tweets posted in the second week of March (Fig 3).

### 3.2 Misinformation by location

The total number of tweets, misinformation and misinformation ratio is found in Table 2 for countries with at least 24,000 posted tweets and for the rest of countries in S2 Table. Since English keywords were used, the majority of collected tweets in the present study was obtained from English speaking countries such as the US, UK, India, Ireland, Canada, Australia, and Nigeria. The content generated by users in the US constituted about 17% of the total content used during the study. The ratio of misinformation tweets was calculated by dividing the number of misinformation tweets by the total number of tweets for each country. The results showed that most Asian countries (e.g., Japan, South Korea, and Singapore) had a low ratio of misinformation (4%), and South Africa and European countries such as Spain, Ukraine, Netherlands, and France had a higher ratio (7%).

**Table 2:** Vaccine-related tweets per country.

country	#tweets	#misinformation	Misinformation ratio
US	1,253,299	85,232	6.8%
UK	336,158	22,389	6.7%
India	197,729	12,690	6.4%
Canada	196,110	12,952	6.6%
Australia	45,201	2,760	6.1%
Nigeria	43,533	2,673	6.1%
Ireland	34,701	2,337	6,7%
South Africa	30,358	2,111	7%
France	24,413	1,825	7,5%

**Fig 4:**
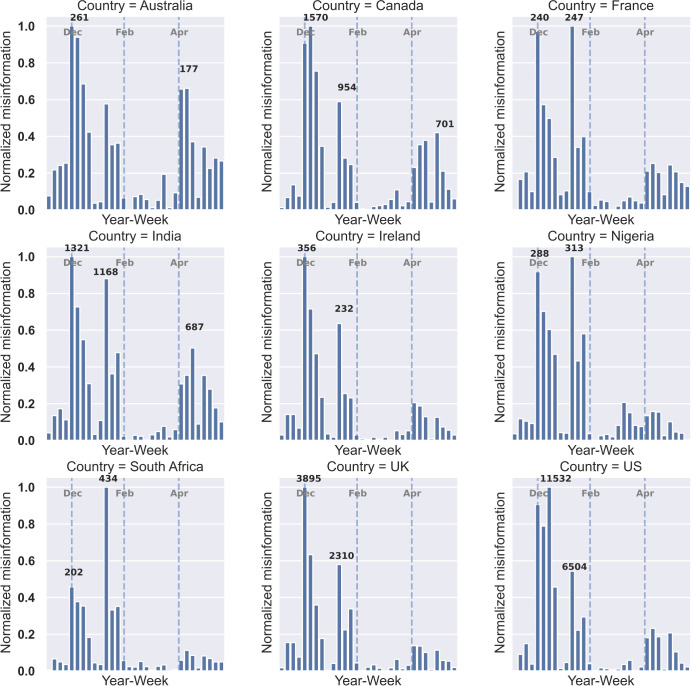
Weekly normalized misinformation per country. x-axis showing the week numbers of 2020 and 2021 and y-axis showing the amount of misinformation that is normalized between 0 and 1 using the popular Min-Max normalization technique. In addition, we annotated the high peaks of plots with the actual numbers of misinformation tweets. Further, we indicated months of December, February, and April with vertical dashed lines in each plot.

Fig 4 depicts the progression of misinformation during the present study for the countries with the most posted tweets. In general, the dissemination of misinformation was higher at the start of the study duration, especially in December and January. The highest amount of misinformation was spread in the US where the number of misinformation tweets reached 11,532 tweets in December and 6,504 tweets in January. While some countries such as the US, the UK, India, Australia, Canada, and Ireland had a higher number of misinformation tweets in December, others like Nigeria, South Africa and France had higher peak of misinformation in January.

### 3.3 Demographic and engagement metrics of misinformation-spreaders

We explored the impact of the account’s demographics and characteristics on the spread of misinformation. This can be valuable in determining which accounts were responsible for spreading vaccine misinformation. A M3-inference model was used to infer demographic attributes. A python script was developed to fetch accounts’ information from the user table and write them as a JSON list file. The list included user information including usernames and profile image addresses. The JSON input file was then passed to a M3-inference model to infer a JSON output file indicating the probability of demographic categories for each user. The value for a particular attribute was selected by choosing the highest probability group compared to other groups. For example, an account with the highest probability for the age group of under 18 years old falls into the first age category.

**Fig 5:**
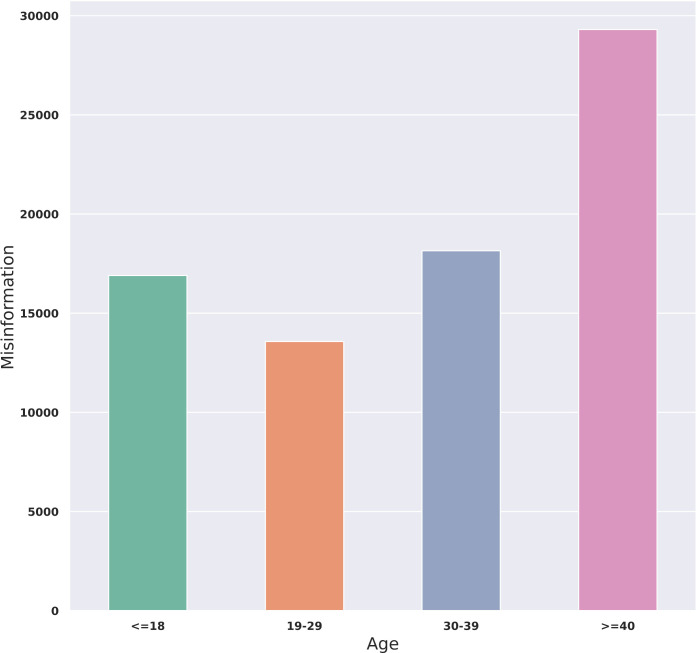
Distribution of misinformation by age group.

The amount of misinformation based on age group of accounts is shown in Fig 5. The highest proportion of misinformation was posted by the population over the age of 40, followed by 30 to 39 years old with around 29,000 and 18,000 tweets respectively. The lowest amount of misinformation was generated by the users aged between 19 and 29 whose spread misinformation content was half of the amount that the eldest group posted.

**Fig 6:**
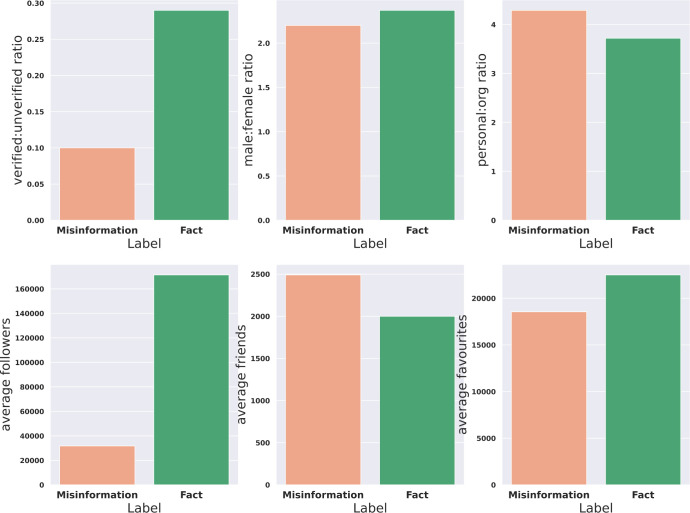
Comparing demographics/engagement of misinformation and fact spreaders’ accounts. Comparison between fact and misinformation in terms of ratio of verified to unverified accounts (top left), ratio of male to female accounts (top middle), ratio of personal to organization accounts (top right), average followers of accounts (bottom left), average friends of accounts (bottom middle), average favourites of accounts (bottom right).

Demographic attributes including verification status (top row, first column), gender of accounts (top row, second column) and organization status (top row, third column) were compared in facts and misinformation content (Fig 6). Twitter assigns a verified status to real-world entities such as academic institutions, but most Twitter accounts are individual accounts and unverified [39]. Furthermore, we averaged the number of followers (bottom row, first column), friends (bottom row, second column), and favourite hits (bottom row, third column) in facts and misinformation content (Fig 6). To make a fair comparison among demographic groups, with different populations, we calculated the ratio metrics. The ratio of males to females was slightly lower in misinformation content, which means males spread less misinformation. Twitter users spreading misinformation include more individual accounts than organizational accounts and had three times less verified badges than users spreading accurate information. Moreover, compared to those circulating accurate information, misinformation spreaders had given 27% less favourite hits and were followed by five times less people, but had made 25% more friends.

### 3.4 Bot-like activities

Bot-like activities in Twitter accounts were explored to understand the role of automated accounts in the spread of misinformation regarding COVID-19 vaccines. The Botometer API was authenticated using Twitter API credentials and RapidAPI key and was called to find bot activities. We queried a random sample of 1,000 tweets from user accounts that posted tweets with class labels of misinformation and facts. Subsequently, the accounts’ screen names were sent as input for the “check accounts in” function of the Botometer.

**Fig 7:**
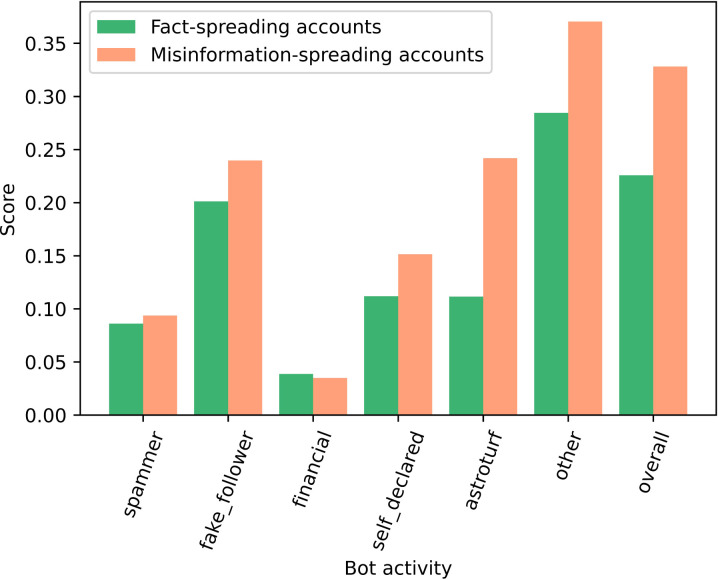
Comparing misinformation-spreading and fact-spreading accounts in terms of their bot-like activities. x-axis shows bot-like activities and y-axis shows the probability of each activity. In the plot, the accounts corresponding with misinformation content are show in orange and the fact-spreading accounts in green.

We compared samples of accounts in each class label in terms of their bot-like activities in Fig 7. Bot-like activities had an overall higher score among misinformation-spreading accounts. In particular, the average score of Hyper-active political bots (Astroturf) in misinformation spreaders (0.25) was twice of that fact-spreading accounts (0.12). Astroturfing is defined as mimicking ordinary political participation by autonomous individuals [40]. The next highest activity in misinformation spreaders accounts compared to the fact-spreading accounts were ‘selfdeclared’ and ‘fake _follower’ [33] However, the financial and spammer activities were similar in both groups of accounts.

## 4. Discussion and conclusions

Vaccine-related misinformation on Twitter can cause vaccine hesitancy and consequently might influence individuals to refrain or delay receiving a COVID-19 vaccines [41]. Therefore, it is important to public health authorities to identify and to better understand misinformation sources spreading through social media.

The approach used in the present study to predict vaccine misinformation helps reduce the time and cost of labeling. The use of active learning approach is a contribution to the performance of misinformation prediction by involving domain experts in preparing the initial labeled dataset and also to do it in an interactive environment.

An increase in misinformation was noted during important events such as vaccine emergency approvals, calls for a pause of vaccines due to safety concerns, and issuing vaccine passports. For example, the increase in misinformation tweets in April and May, 2021 could be regarding the discussions around the risk of taking AstraZeneca and Johnson & Johnson vaccines and vaccine passport concerns. It is likely that those who want to spread misinformation see an opportunity when an important event takes place in the issue of vaccines. People will be more in tuned with vaccine related information and therefore will seek to become more informed. Those that pedal in misinformation will see this opportunity to misinform and further their cause. In support of this notion, misinformation reached its lowest amount during February and March, 2020. which could be due to news regarding vaccine acceptance among American and two doses of the Pfizer Vaccine being 98.8% effective against deaths and hospitalization in February.

Our study showed a lower ratio of misinformation in Asian countries such as Japan, South Korea and Singapore compared to other countries. The low amount of misinformation in Asian countries was consistent with a previous study [42]. It is important to note that considering the low number of English tweets collected for the non-English speaking counties, their ratio of misinformation can be biased.

Extracted demographic information revealed the largest amount of misinformation posted by the Twitter users over the age of 40, followed by the age range between 30 and 39. This was aligned with other studies showing younger people are more willing to receive vaccines than older individuals [24, 43–45]. This could be explained by those individuals that were vaccine hesitant or less likely to receive a vaccine, and are more apt to share misinformation about the hazards of COVID-19 vaccines among their followers. However, there is a possibility of biased results in the classification of demographic attributes through the M3inference API. The span of age groups was not divided equally. For example, the oldest group had the largest span and included those who were over 40 years old while the middle-aged group was defined as ages between 30 to 39.

Our findings showed a lower ratio of males to females in misinformation content than fact content, showing males having more confidence in vaccines. Previous studies also found males to be more willing to take vaccines while females had more negative opinions about it [5, 44, 46, 47]. Again supporting the premise that those who are more likely to be vaccine hesitant are more likely to share this information that supports their view.

We found individual Twitter accounts contributed more tweets than organization accounts to spreading misinformation. This is not surprising as organizational accounts posting in favour of COVID-19 vaccines are usually associated with well-known news media outlets and public health agencies [10]. Our findings also showed user accounts spreading misleading information had received less verified badges than users spreading accurate information.

Comparing demographics and other characteristics of Twitter users showed that misinformation spreaders tried to make a stronger network by following more people, while users sharing facts were more active in giving favourite hits and were followed by more users. This was also evident in our analysis identifying the likelihood of bot-like activities in both fact-sharing and misinformation spreader groups, where the former group showed a higher probability of having fake followers.

Detection of misleading vaccine-related tweets and accounts spreading them can help improve vaccine acceptance. Misinformation content can include tags showing the possibility of carrying misinformation so that Twitter users would be warned and avoid reading or sharing them. Similarly, suspicious accounts need to be identified in some ways by Twitter.

The amount of misinformation used in the present study was only around 6% of total collected tweets. The reason behind the low amount of misinformation after December 2020 could be the effort that Twitter has made to protect the public against COVID-19 misinformation. Since late December 2020, Twitter had prioritized the removal of the most harmful misleading information and challenged the associated accounts [48]. Further, beginning March 2021, Twitter has begun to label Tweets that contain potentially misleading information about the vaccines.

While the policy is a step in the right direction, 6% misinformation means that misinformation about COVID-19 vaccines was still circulating during the time window of the present study. A representative sample of US adults found that misinformation is widespread, with 85% believing at least one conspiracy theory [49]. The spread of misinformation creates confusion among the public with regards to which sources are trustworthy [22].

Several factors such as random sampling, the division boundaries of age groups, and other socio-demographic factors such as wealth, education, and race might have influenced our findings. Moreover, although English is a widely spoken language on Twitter, the geographical insights derived from the current study can be biased as only English-language tweets were collected. Furthermore, the profile locations defined by Twitter users cannot necessarily indicate the actual locations that tweets were posted from. Despite our efforts to standardize the location names, we might have missed or been unable to map some of them with a country name.

The dissemination of information on social media should be closely monitored by policymakers for future COVID-19 mass vaccination efforts to ensure its uptake. In the current study, we built a machine-learning model to identify misinformation tweets. The study found novel insights regarding COVID-19 vaccine misinformation spread such as critical time points, geographical impact, and spreaders’ account characteristics such as their demographic, engagement metrics and bot activities. The snapshot of misinformation patterns in this study might be used for controlling the spread of misleading information by targeting a specific demographic or putting the focus on critical information in important points.

Future research might categorize Twitter accounts to those that have been deliberately spreading misinformation and those that may spread misinformation with no intention. The former group then can be suspended or given warnings and the latter group can be targeted for education. Moreover, future research may develop real-world applications by creating indicators that can be used as a benchmark for social media networks to prevent the spread of false, and potentially harmful, information.

## Supporting information

S1 Table Labeling guidelines. (DOCX)

S2 Table Vaccine-related tweets per country (for countries with less than 24,000 tweets). (DOCX)
