# Supplementary material for: Credibility of vaccine-related content on Twitter during COVID-19 pandemic
Source: PLOS Glob Public Health. 2023 Jul 19;3(7):e0001385. doi: 10.1371/journal.pgph.0001385 (PMC10355402; doi:10.1371/journal.pgph.0001385)
Supplement: S1 Table — (DOCX) [file pgph.0001385.s001.docx]

S1 Table: Labeling Guidelines

| misinformation | non-misinformation |
| --- | --- |
| Suggesting treatments without any credible source of information: for example, ”taking Zink is more effective than experimental vaccines” or ”I don’t need vaccine because I already got COVID and I have anti-bodies”.  Blames about vaccine consequences without a credible source: for example, ”Moderna vaccine can mess you up”  Conspiracy theories and myths such as containing 5G microchips, poison, and fetal tissues, or causing infertility, magnetic property, abd autism. vaccines are not tested and won’t work. vaccine causes long-term complications.  If there is a doubt, check if the links in the text are from legitimate websites such as CNN. The credibility of tweets can be checked using tools such as *website authority checker* and *scam adviser*. | individuals’ vaccination experience.  hopes and prayers for the end the pandemic with vaccines. advertisement. political issues.  Guiding anti-vaxxers about vaccine safety.  Reports on COVID-19 cases/deaths and vaccine distributions.  scientific articles |
