# Supplementary material for: Credibility of vaccine-related content on Twitter during COVID-19 pandemic
Source: PLOS Glob Public Health. 2023 Jul 19;3(7):e0001385. doi: 10.1371/journal.pgph.0001385 (PMC10355402; doi:10.1371/journal.pgph.0001385)
Supplement: S2 Table — (DOCX) [file pgph.0001385.s002.docx]

S2 Table: Vaccine-related tweets per country (for countries with less than 24,000 tweets)

| country | #tweets | #misinformation | Misinformation ratio |
| --- | --- | --- | --- |
| Spain | 23,129 | 1,572 | 6.8% |
| South Korea | 22,710 | 885 | 3.9% |
| Ukraine | 20,501 | 1,496 | 7.3% |
| Netherlands | 19,133 | 1,454 | 7.6% |
| Singapore | 15,432 | 617 | 4% |
| Japan | 12,897 | 70 | 3.7% |
|  |  |  |  |
